# Roles of telomeres and telomerase in cancer, and advances in telomerase-targeted therapies

**DOI:** 10.1186/s13073-016-0324-x

**Published:** 2016-06-20

**Authors:** Mohammad A. Jafri, Shakeel A. Ansari, Mohammed H. Alqahtani, Jerry W. Shay

**Affiliations:** Center of Excellence in Genomic Medicine Research, King Abdulaziz University, Jeddah, 21589 Kingdom of Saudi Arabia; Department of Cell Biology, University of Texas Southwestern Medical Center, Dallas, Texas 75390 USA

## Abstract

Telomeres maintain genomic integrity in normal cells, and their progressive shortening during successive cell divisions induces chromosomal instability. In the large majority of cancer cells, telomere length is maintained by telomerase. Thus, telomere length and telomerase activity are crucial for cancer initiation and the survival of tumors. Several pathways that regulate telomere length have been identified, and genome-scale studies have helped in mapping genes that are involved in telomere length control. Additionally, genomic screening for recurrent human telomerase gene *hTERT* promoter mutations and mutations in genes involved in the alternative lengthening of telomeres pathway, such as *ATRX* and *DAXX*, has elucidated how these genomic changes contribute to the activation of telomere maintenance mechanisms in cancer cells. Attempts have also been made to develop telomere length- and telomerase-based diagnostic tools and anticancer therapeutics. Recent efforts have revealed key aspects of telomerase assembly, intracellular trafficking and recruitment to telomeres for completing DNA synthesis, which may provide novel targets for the development of anticancer agents. Here, we summarize telomere organization and function and its role in oncogenesis. We also highlight genomic mutations that lead to reactivation of telomerase, and mechanisms of telomerase reconstitution and trafficking that shed light on its function in cancer initiation and tumor development. Additionally, recent advances in the clinical development of telomerase inhibitors, as well as potential novel targets, will be summarized.

## Background

Cancer is generally an age-related genetic disease, manifesting only when normal cells accumulate genomic instability over a period of time and acquire the capability of replicative immortality. Telomere attrition during successive cell divisions induces chromosomal instability and contributes significantly to genomic rearrangements that can result in tumorigenesis. Telomeres, repetitive (TTAGGG) DNA–protein complexes at the ends of chromosomes, are crucial for the survival of cancer cells. They are maintained by an enzyme called telomerase in the vast majority of tumors. The mechanisms underlying telomere length (TL) maintenance and telomerase expression involve transcriptional, post-transcriptional and epigenetic regulation, and in-depth understanding of these mechanisms may provide novel biomarkers and targets for early detection of disease, determination of disease prognosis, and the development of therapeutics [1].

Telomeres protect chromosome ends from fusion and from being recognized as sites of DNA damage (Box 1). Dysfunctional telomeres, arising by critical shortening of telomeres in normal somatic cells during progressive cell divisions, elicit DNA damage responses (DDRs) that trigger cellular senescence. Cells that gain oncogenic changes bypass senescence and continue to divide (extended lifespan period) until multiple critically shortened telomeres initiate crisis (a period of complete replicative senescence, chromosome end-to-end fusions, and extensive apoptosis). This leads to breakage–fusion–bridge cycles in which two sister chromatids lacking telomeres fuse together, forming a bridge with a chromatin connection. During anaphase, the sister chromatids are drawn apart owing to movement towards opposite poles, resulting in the formation of uneven derivative chromosomes, leading to genomic instability. The period of crisis results in extensive cell death. However, certain rare cells escape crisis and maintain stable but usually shortened telomere lengths for continued cell growth, eventually progressing to a malignant phenotype. Cancer cells achieve proliferative immortality by activating or upregulating the normally silent human *TERT* gene (*hTERT*) that encodes telomerase, a protein with reverse transcriptase activity that complexes with other proteins and a functional RNA (encoded by *hTR*, also called *hTERC*) to make a ribonucleoprotein enzyme complex. Rarely, another DNA recombination mechanism termed alternative lengthening of telomeres (ALT) reverses telomere attrition in order to bypass senescence. Although *hTERT* is usually silenced in almost all somatic cells, it is significantly expressed in ~90 % of human cancers. The details of the underlying mechanisms of *hTERT* activation are still being elucidated, but they mainly include mutations in the *hTERT* promoter, alterations in alternative splicing of *hTERT* pre-mRNA, *hTERT* amplification, epigenetic changes, and/or disruption of telomere position effect (TPE) machinery [2].

Recent reports have implicated two cancer-specific *hTERT* promoter mutations (mainly C˃T transitions) in the activation of telomerase in cancer cells [3, 4]. These mutations, which are located either −124 base pairs (bp) or −146 bp upstream from the TERT translation start site [5, 6], have been found to be associated with increased telomerase activity [7]. Therefore, molecular mechanisms that regulate *hTERT* expression and telomerase assembly have been subjected to intense investigation. Studies using telomerase inhibition strategies have established that robust *hTERT* inhibition can lead to progressive telomere shortening and eventually cancer cell death. Several approaches, including use of small-molecule inhibitors, antisense oligonucleotides, immunotherapy, and G-quadruplex stabilizers have been employed to inhibit telomerase function [8]. Currently, many anti-telomerase therapeutics are being evaluated in clinical trials against a variety of cancer types. The following sections will cover recent developments in the area of telomere and telomerase biology, their implications for understanding mechanisms underlying cancer and for the development of cancer therapies, as well as outstanding questions for the field.

## Telomeres: organization, function and association with cancer

**Fig. 1:**
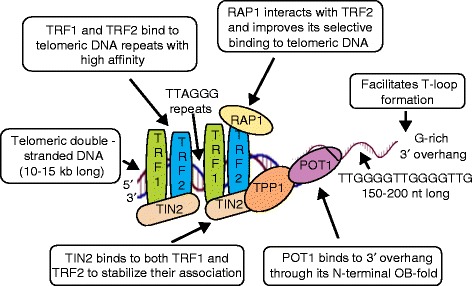
Schematic representation of telomeric DNA and components of the shelterin complex. Telomeres comprise a specialized nucleoprotein-capping structure consisting of DNA and shelterin protein complexes. Telomeric DNA contains a variable number of G-rich, non-coding, tandem repeats of the double-stranded DNA sequence 5′-(TTAGGG)_*n*_-3′, followed by a terminal 3′ G-rich single-stranded overhang (150–200 nucleotides (*nt*) long). The 3′ G-rich overhang facilitates telomeric DNA in forming a higher-order structure in which the 3′ single-stranded overhang folds back and invades the homologous double-stranded TTAGGG region, forming a telomeric loop (*T-loop*) that provides 3′-end protection by sequestering it from recognition by the DNA damage response machinery. The proteins associated with telomeres form the shelterin complex, which consists of three core shelterin subunits, TRF1 and TRF2, which directly recognize and bind duplex TTAGGG repeats, and POT1, which recognizes and binds single-stranded TTAGGG overhangs. These three proteins are interconnected by three additional shelterin proteins, TIN2, TPP1 and RAP1, forming a complex that enables the DNA damage response surveillance machinery to distinguish telomere DNA from sites of genomic DNA damage

Recent studies have significantly contributed to our understanding of telomere organization in the nucleus, telomere profiling for risk stratification, and the signaling pathways that mediate modulation of telomere structural component proteins or factors to regulate gene transcription [9]. Telomeres consist of a capping structure, which is a specialized nucleoprotein structure consisting of DNA and shelterin protein complexes. Telomeric DNA contains a variable number of G-rich, non-coding, tandem repeats (10–15 kilobases (kb) long in humans at birth) of double-stranded DNA sequence, 5′-(TTAGGG)_*n*_-3′, followed by a terminal 3′ G-rich single-stranded overhang (150–200 nucleotide long). The 3′ G-rich overhang facilitates telomeric DNA in forming a higher-order structure in which the 3′ single-stranded overhang folds back and invades the homologous double-stranded TTAGGG region, forming a telomeric loop (T-loop) that provides 3′-end protection by sequestering it from recognition by the DDR machinery [10]. The proteins associated with telomeres are called the shelterin complex, which consists of three core shelterin subunits, TRF1 and TRF2, which directly recognize and bind duplex TTAGGG repeats, and POT1, which recognizes and binds single-stranded TTAGGG overhangs. These three proteins are interconnected by three additional shelterin proteins, TIN2, TPP1 and RAP1, forming a complex that enables DDR surveillance machinery to distinguish telomere DNA from sites of genomic DNA damage (Fig. 1). The shelterin complex performs critical and distinct functions that ensure telomere stability. For example, TRF2 is required for T-loop formation and maintenance of ATM-mediated DDR suppression and repression from non-homologous end joining [11]. TRF1 has a central role in controlling replication of telomeric DNA [12] while POT1 associates with TPP1 to bind the single-stranded 3′ overhang and repress ATR-mediated DDR by preventing the recruitment of replication protein A (RPA) [13]. TIN2 is essential to the overall integrity of the shelterin complex as it links the TPP1/POT1 heterodimer to TRF1 and TRF2, and stabilizes TRF1 and TRF2 associations with telomeric DNA [14, 15]. RAP1 interacts with TRF2 and improves its selective binding to telomeric DNA [16].

Apart from DNA end protection, telomeres also perform other important functions such as regulation of gene expression through transcriptional silencing of genes located close to the telomeres, called TPE [17], or located at long distances from telomeres, termed TPE over long distances (TPE-OLD) [18]. The function of telomeres is tightly regulated and depends on a minimal length of telomeric repeats and the functionality of the associated shelterin protein complexes. In addition, higher-order DNA conformations, such as the T-loop and G-quadruplexes (G-rich four-stranded non-helical structures) are thought to contribute to normal telomere function. Moreover, telomeric chromatin has an important role in telomere maintenance, signaling and regulation of telomere function, but many of the precise structures and molecular mechanisms of human telomeric chromatin are not well understood. However, telomeric regions contain telomeric repeat-containing RNA (TERRA), a long non-coding RNA that is transcribed from telomeric DNA by RNA polymerase II [19]. TERRA has been implicated in telomerase regulation, organization of heterochromatin at telomeres, regulation of gene expression, and in DDR triggered by dysfunctional telomeres [19]. The mammalian cell lines harboring active ALT have higher TERRA levels compared with telomerase-positive cells [20]. However, the exact role of TERRA in activation of the ALT mechanism is not clear [21].

**Fig. 2:**
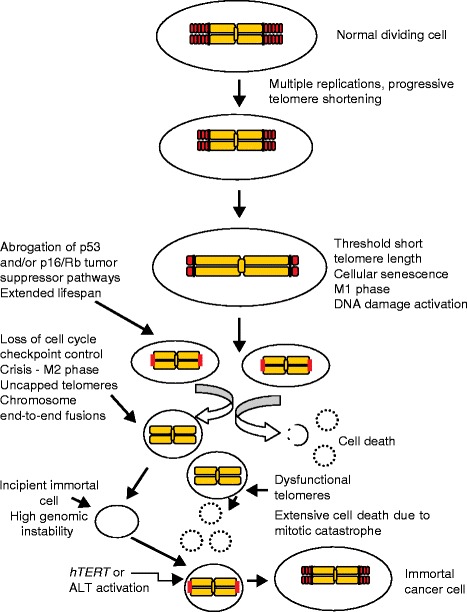
Cellular senescence and crisis. Telomeres protect chromosome ends from undergoing fusions and recombination by masking telomeric DNA with shelterin protein protective caps, preventing the ends from being recognized by the DNA damage surveillance pathways. Telomere shortening is a natural consequence of cell division due to the “end replication problem” whereby lagging strand DNA synthesis cannot be completed all the way to the very end, and increased cell divisions lead to critically shortened telomeres which elicit DNA damage responses that trigger cellular senescence. In the cells undergoing replicative senescence, the p53 and p16–RB pathways are often activated leading to essentially irreversible growth arrest. Cells that gain additional oncogenic changes (p53 loss) can bypass senescence and continue to divide until multiple critically shortened telomeres initiate crisis, a period of increased chromosome end-to-end fusions and extensive cell death. Only a rare human cell (one in 10^5^ to 10^7^) can engage a mechanism to bypass crisis and become immortal. This is almost universally accomplished by the upregulation or reactivation of telomerase. A rarer telomerase negative immortalization pathway, termed ALT (alternative lengthening of telomeres), involves DNA recombination to maintain telomeres

TL is critically important in normal cells, and telomere shortening can—in combination with other oncogenic changes—promote genome instability, potentially stimulating initiation of the early stages of cancer. In humans, the distribution of TL among different chromosome arms is heterogeneous. TL reduces at a rate of 50–150 bp at each cell division in human somatic cells in cell culture. Consequently, individual telomere shortening rates may be different in different cell lineages. The time point at which any chromosome end will become uncapped depends on the specific TL shortening rate in each cell type or tissue. Thus, the shortest telomere is critically important for cell viability and chromosomal stability as it may be a sole contributor to the senescence onset signal [22]. There are two critically important barriers that prevent cell immortalization and ultimately malignant transformation: replicative senescence and crisis [23]. The period of cellular senescence, also known as mortality stage 1 (M1), is characterized by inhibition of cellular proliferation, probably due to the uncapping of one or a few shortened telomeres. In the presence of cancer-initiating changes, M1 can be bypassed, providing an extended cell division period. However, during this phase additional telomeres become very short and these “marked” telomeres result in a new dysfunctional state, termed crisis (or M2 crisis). M2 is a period in which signals to undergo replicative senescence and signals for cells to continue to divide are balanced. This eventually results in chromosome end-to-end fusions and extensive cell death (apoptosis) [24]. However, a rare clone (1 in 100,000 to 1 in 10 million cells) can progress towards the acquisition of cell immortality [25]. At this point, a mechanism must be engaged to maintain these very short telomeres, and this occurs by either increasing or reactivating telomerase expression, or by acquiring a much rarer telomerase-independent ALT mechanism, thus bypassing crisis and ultimately leading to cell immortalization [26] (Fig. 2).

Although telomerase maintains telomere length in the majority of cancer cells, the ALT mechanism is also employed by 10–15 % of tumors [27]. The ALT pathway utilizes a homologous recombination-based DNA replication mechanism to extend telomere length. The activation of the ALT mechanism is thought to involve loss of chromatin-remodeling factors such as ATRX and DAXX, resulting in reduced compaction of telomeric chromatin, which leads to the production of altered telomeric DNA sequences and activation of a telomere-specific DDR pathway [28, 29], which in turn stimulates homology-directed synthesis of telomeric DNA. Recently, Flynn and colleagues [30] reported that inhibition of the protein kinase ATR disrupts the ALT mechanism in ALT-positive cancer cells, resulting in cell death. This suggests that ATR inhibitors may be a useful therapeutic intervention for ALT-harboring tumors.

## Telomerase: the key telomere length maintenance mechanism

Telomerase is a large ribonucleoprotein complex responsible for progressive synthesis of telomeric DNA repeats (TTAGGG) at the 3′ ends of linear chromosomes, thereby reversing the loss of DNA from each round of replication. Telomerase is a reverse transcriptase that consists of a catalytic protein subunit called telomerase reverse transcriptase (TERT), encoded by the *hTERT* gene in humans that is positioned at chromosome 5p15.33, and an essential RNA component known as human telomerase RNA (hTR) or human telomerase RNA component (hTERC), encoded by the *hTERC* gene found on chromosomal region 3q26. hTR acts as a template (carries sequence complementary to one or more copies of telomeric repeats) for the synthesis of telomere DNA, and is also involved in the catalysis, localization and assembly of the telomerase holoenzyme [31]. Recent studies have reported that, in addition to TL maintenance, telomerase is also involved in gene expression regulation, cell proliferation, apoptosis, WNT/β-catenin signaling, NF-kB signaling, MYC-driven oncogenesis, DDR, cell adhesion and migration, and epithelial–mesenchymal transition [32–35]. All these activities of telomerase are thought to contribute significantly to the process of oncogenesis.

TL maintenance by telomerase is a complex multistep process that involves a series of molecular events including hTERT protein transport and trafficking into the nucleus, hTR and hTERT assembly with accessory components in the nucleus, and recruitment to telomeres at the appropriate time during DNA replication. It has been reported that at least hTERT and hTR are essential for the in vitro reverse transcriptase activity of the human telomerase enzyme [36]. However, under in vivo conditions the telomerase holoenzyme also contains four additional proteins—dyskerin, NHP2, NOP10 and GAR1 (localization factor)—associated with the H/ACA class of small nucleolar RNAs that play an important role in the process of pseudouridylation during post-transcriptional modification of RNAs. In addition, a WD-repeat-containing protein 79 called TCAB1 binds to the CAB-box sequence within hTR and directs the telomerase holoenzyme to localize at Cajal bodies bound to the nucleolus [37]. Numerous additional factors such as the chaperones HSP90 and p23, as well as the ATPases pontin and reptin, have also been observed to bind to the two main subunits of telomerase [38]. Many of these factors are thought to be involved in the assembly of a functional telomerase holoenzyme in vivo but the actual mechanisms by which they interact with telomerase remain poorly understood. One working model of human telomerase biogenesis is that dyskerin, pontin and reptin form a scaffold and create an assembly platform for nascent hTR transcripts. Then, the H/ACA motif-binding complex of dyskerin, NHP2 ribonucleoprotein, NOP10 ribonucleoprotein, a nuclear assembly factor ribonucleoprotein (NAF1) and the telomerase ribonucleoprotein (RNP) particle associate. Next, hTR removes NAF1 and attaches GAR1, leading to the formation of a physiologically stable hTR-H/ACA-RNP complex. The hTR 3′-hairpin CAB-box sequence recruits TCAB1, and finally hTERT binds to two structurally independent hTR domains (CR4/CR5), thus generating the catalytically active telomerase RNP [39]. TCAB1, found in Cajal bodies, binds to the CAB box of hTR and guides telomerase to the Cajal bodies, where it remains localized for most of the cell cycle, but the physiological significance of this process is not known.

**Fig. 3:**
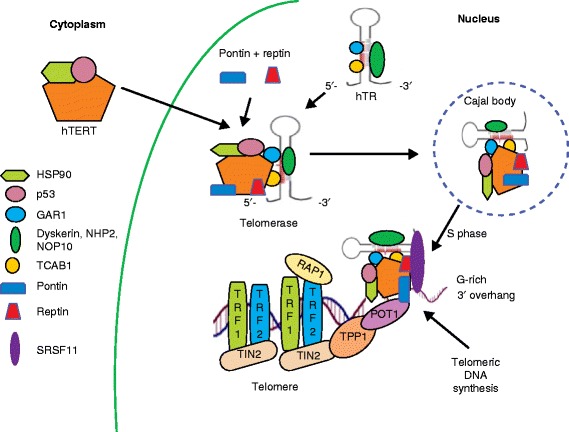
Telomerase assembly, recruitment to the telomere, and telomeric DNA synthesis. Telomerase is the cellular ribonucleoprotein enzyme complex that catalyzes the extension of telomeric DNA in eukaryotic organisms. Telomerase action involves multiple steps including assembly of the telomerase complex, its intracellular trafficking and finally recruitment to telomeres. Human telomerase is composed of hTR (hTERC—a template functional RNA), hTERT (the catalytic protein component with reverse transcriptase activity), and the accessory proteins dyskerin, NOP10, NHP2, and GAR1. hTERT protein associates with p23 and HSP90 in the cytoplasm, and moves to the nucleus. Nascent hTR transcripts complex with dyskerin, NHP2, NOP10 and GAR1. This complex then undergoes Reptin and Pontin (ATPases)-mediated binding to hTERT + p23 + HSP90 complex. Then TCAB1 attaches to this assembling complex and guides it to Cajal bodies in the nucleus. Telomerase recruitment to telomeres takes place during the S phase of the cell cycle through interactions between the shelterin complex components TPP1 and POT1 and the DAT domain of hTERT. SRSF11 stabilizes the association of the telomerase enzyme complex with the telomere overhang for DNA synthesis

The recruitment of telomerase to telomeres occurs only after the replication fork remodels the protected DNA 3′ ends during the S phase of the cell cycle. It involves protein–protein interactions between the shelterin complex components TPP1 and POT1 and the DAT (dissociates the activities of telomerase) domain of hTERT, a region that differentiates the in vivo functionality of hTERT from its in vitro activity. TPP1 contains an N-terminal oligonucleotide/oligosaccharide-binding (OB)-fold domain that includes a patch of amino acids termed the Tel patch, which directly interacts with the telomerase DAT domain [40]. It also contains a central domain that directly binds to POT1 and a C-terminal domain that associates with TIN2. Thus, interaction between the DAT domain of hTERT and shelterin components ensures correct positioning of telomerase at the 3′ end of DNA for synthesis and processivity of telomeric repeats. Telomerase loading onto the telomeres is mediated by SRSF11 (a novel TERC-binding protein), which leads to the stable association of the enzyme with the telomere overhang, and proper positioning of the DNA 3′ end at the active site of the enzyme for nucleotide addition [41] (Fig. 3).

## The role of telomerase in cancer: *TERT* promoter mutations and telomerase reactivation

Telomerase upregulation or reactivation is a critical feature in over 90 % of cancers. However, the mechanisms governing *hTERT* expression in cancer remain incompletely understood. Therefore, understanding how *hTERT* is activated in cancer cells and how it contributes to further progression of the disease continues to be a major area of research.

*hTERT* is a 40 kb gene consisting of 15 introns and 16 exons. It is located on the short arm of human chromosome 5 (5p15.33) approximately 1.2 megabases away from the telomere, embedded in a nuclease-resistant chromatin domain [42]. The *hTERT* promoter is GC rich and lacks both TATA (found in the promoter regions of genes that encode proteins found in both eukaryotes and prokaryotes) and CAAT (which rarely occurs in the promoter region of eukaryotes but is completely absent in prokaryotes) boxes but contains binding sites for multiple transcription factors, suggesting that *hTERT* expression is under multiple levels of control and may be regulated by different factors in different cellular contexts.

**Fig. 4:**
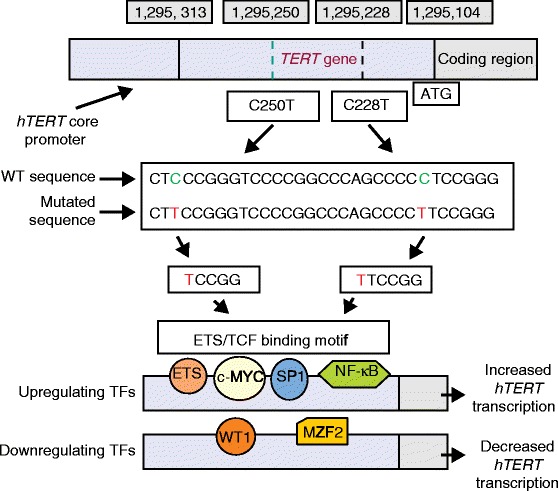
*hTERT* transcription and promoter mutations. The *hTERT* gene is tightly repressed in almost all normal cells and tissues. Specific *hTERT* promoter mutations as part of cancer progression occur leading to increased transcription of *hTERT*. The transcription of *hTERT* is regulated by a series of transcription factors (*TFs*). *hTERT* promoter mutations create ETS/TCF binding motifs. Each mutation generates a new ETS/TCF binding site. Upregulating TFs such as ETS, c-MYC, SP1 and NF-kB bind to their respective sites and can promote *hTERT* transcription. Although binding of TFs is essential for *hTERT* transcription, in addition a permissive chromatin microenvironment is required. Binding of downregulating transcription factors decreases transcription. *WT* wild type; *WT1* Wilms tumor protein1; *MZF2* myeloid zinc finger protein 2

The 260 bp proximal region designated as the *hTERT* promoter core is responsible for most of its transcriptional activity. It contains at least five GC boxes (GGGCGG), which are binding sites for the zinc finger transcription factor SP1, and are essential for *hTERT* promoter activity. Two E-boxes (5′-CACGTG-3′), located at positions −165 and +44 of the nucleotide sequence of *hTERT* relative to the transcription start site (TSS), provide binding sites for several enhancer binding proteins such as the MYC/MAX/MXD1 family and USF1/2. The E-boxes are not only important for *hTERT* promoter activation by c-MYC, but also bind to MAD1 and USF1 to mediate *hTERT* repression. The *hTERT* promoter core also contains a single TSS that binds the multifunctional transcription factor TFII-I. The transcription of the *hTERT* promoter is regulated by the action of multiple transcription factors and the telomere chromatin environment. However, it remains unsolved how the interplay between transcription factors and the telomere chromatin milieu controls *hTERT* transcription. Several transcription factors bind to the *hTERT* promoter core to activate or repress *hTERT* transcription. The transcription factors that upregulate transcription include c-MYC, SP1, E-twenty-six (ETS) family members, NF-kB, AP-2 and HIF-1. Transcription factors such as p53 (also known as TP53; represses transcription in an SP1-dependent manner), MAD (transcription factor involved in a network controlling cell cycle progression), WT1, MZF-2, SIP1 and menin have been shown to downregulate *hTERT* transcription. Most of the transcription factors that upregulate the telomerase gene are widely expressed and cannot fully account for high levels of *hTERT* expression and activation during tumorigenesis (Fig. 4).

Recent observations of two highly recurrent mutations at two sites within the core promoter region of *hTERT* suggest one possible mechanism for the activation of telomerase in cancer cells. These mutations, which occur at −124 bp and −146 bp upstream from the ATG start site, are C˃T transitions (at positions 1,295,228 (C228T) and 1,295,250 (C250T) on chromosome 5), and each mutation generates an identical 11 bp nucleotide stretch (5′-CCCCTTCCGGG-3′) containing a consensus binding motif (GGA(A/T)) for ETS transcription factors that can function as transcriptional repressor, activator or both to regulate telomerase expression [3, 4]. However, the molecular mechanisms of telomerase activation by ETS are not clearly understood. It has recently been reported that epidermal growth factor (EGF)-mediated activation of telomerase activity in lung cancer is associated with direct binding of ETS-2 to the *hTERT* promoter [43]. The recurrent *hTERT* promoter mutations were first reported as germline mutations from a family of melanoma patients and were later seen through genome sequencing of sporadic melanoma (in >74 % melanomas) and a number of cell lines across numerous cancer types and were associated with increased *hTERT* promoter activity [3, 4]. These mutations occur in approximately 70 % of melanomas, 80–90 % of glioblastomas, 60 % of hepatocellular carcinomas, 60 % of bladder cancers, 70 % of basal cell carcinomas, 50 % of cutaneous squamous cell carcinomas, up to 30 % of thyroid cancers and approximately 72 % of oligodendrogliomas [4, 6, 44, 45].

Additionally, a less frequent *hTERT* promoter mutation, −57 bp upstream from the ATG start site, resulting in an A>C transition, and other less frequent but recurrent mutations in cancer are found on chromosome 5 at the following positions: 1,295,228 C>A; 1,295,248–1,295,243 CC>TT; and 1,295,161 A>C [46]. However, when these mutations (−57 A>C, −124 C>T, −146 C>T) are introduced into tumor cells (A375 melanoma cells, UAGCC-62 melanoma cells, T24 bladder cancer cells) using a luciferase reporter construct, only a ~1.5- to 2-fold increase in *hTERT* transcription occurs [4, 45, 47]. Similarly, Huang and colleagues [48] also demonstrated that *hTERT* promoter mutations C228T and C250T caused a 2.8-fold to 5.3-fold increase in transcription (using a luciferase reporter assay in U87-MG cells) and telomerase activation using the telomere repeat amplification protocol (TRAP) assay in both xenografts and primary tumor tissues. It is not clear whether the observed enhanced *hTERT* transcription and increased level of *hTERT* mRNA are actually related to enhanced telomerase functional enzyme activity and TL maintenance in tumor cells.

While *hTERT* promoter mutations are frequent in multiple non-epithelial cancer types and their distribution is similar in the majority of patients, Chiba and co-workers [49] have emphasized that the impact of *hTERT* promoter mutations has mostly been studied in already transformed immortal tumor cells with active telomerase maintaining their telomeres. The tumor cells without such mutations also have sufficient telomerase activity to maintain their telomeres. Therefore, they introduced three common *hTERT* promoter mutations (−57 A>C, −124 C>T, −146 C>T) into isogenic human embryonic stem cells (hESCs) using CRISPR/Cas9 genome editing, and observed that in undifferentiated hESCs the presence of −124 C>T caused a 2- to 3-fold increase in *hTERT* mRNA while neither the −57 A>C nor −146 C>T mutation had any effect on *hTERT* transcription and none of the three mutations had a major influence on telomerase activity. However, differentiated hESCs (fibroblasts) harboring these mutations continued *hTERT* transcription (8- to 12-fold increase) relative to normal hESCs, which would downregulate telomerase activity. Furthermore, telomerase activity in differentiated fibroblasts carrying *hTERT* promoter mutations was comparable to that observed in cancer cell lines. Bell and colleagues [50] proposed that GA-binding protein (GABP), an ETS-binding transcription factor, in conjunction with *TERT* promoter mutations, drives activation of *hTERT*. They have shown that C228T and C250T transitions are necessary for *hTERT* promoter activation, as these generate an ETS motif, which is critically important for the predominant binding of GABP to activate aberrant transcription in cancer cells. However, it is not known whether GABP alone can activate *hTERT* promoter transcription or if it interacts with other ETS-binding transcription factors. Recently, Li and co-workers [51] have pointed out that *hTERT* promoter mutations C228T and C250T are functionally different, in that the C250T unlike the C228T mutation is regulated by non-canonical NF-kB signaling, which is required for sustained telomerase activity.

While these non-coding *hTERT* promoter mutations are the most frequent promoter mutations in cancer, the level and frequency varies with cancer types (Table 1). Some cancers, such as melanoma, pleomorphic dermal sarcoma, myxoid liposarcoma, glioma, urothelial cell carcinoma, carcinoma of the skin and liver cancer, have the highest frequencies of *TERT* promoter mutations, while low frequencies were noted in gastric cancer, pancreatic cancer, non-small-cell lung cancer and gastrointestinal stromal tumors [6, 45, 48]. One possible explanation for these observations could be that incipient cancer cells, originating from rapidly self-renewing telomerase-competent cells, do not require *TERT* promoter mutations to regulate TL maintenance. Thus, cancers arising from these rapidly proliferating cells tend to have less frequent *hTERT* promoter mutations and probably just stably upregulate enzyme activity that is reversibly regulated in normal cells. By contrast, cancer-initiating cells originating from cells with low self-renewing capability may require *TERT* promoter mutations to overcome the short-telomere-dependent proliferative barrier. However, *TERT* promoter mutations have not been detected in prostate cancer, a cancer of low self-renewing tissue, suggesting that alterations within the core promoter of the *TERT* gene do not play an important role in prostate carcinogenesis [52]. The common *hTERT* promoter mutations have been detected across all stages and grades in most cancers, suggesting that *hTERT* mutations are generally an early event in the process of carcinogenesis [49]. It will be interesting to determine whether these mutations mostly occur during the period in which cells are undergoing crisis, in order to establish the role of these mutations as early events in the process of malignant transformation.

**Table 1:** Frequency spectrum of *hTERT* promoter mutations across diverse cancer types

Cancer type	Mutation frequency (%)	Reference
Bladder carcinoma	47–85	[100]
Renal pelvic carcinoma	60–64	[101]
Urothelial carcinoma	47	[102]
Hepatocellular carcinoma	24–59	[6, 103]
Melanoma	67–85	[3]
Skin basal cell carcinoma	39–74	[104]
Thyroid cancer (papillary and poorly differentiated carcinomas)	50–52	[105]
Myxoid liposarcoma	74–79	[106]
Glioblastoma	28–84	[6, 48]
Medulloblastoma	19–42	[107]
Oligoastrocytoma Oligodendroglioma	25–53 72	[6, 107] [44]
Breast cancer, colorectal cancer, medullary thyroid carcinoma, ovarian cancer, esophageal adenocarcinoma, acute myeloid leukemia, chronic lymphoid leukemia, pancreatic cancer, prostate cancer, testicular carcinoma, uterine cervix cancer	0–5	[48, 104]

Telomerase expression also involves transcriptional, post-transcriptional and epigenetic levels of control, which may occur at any critical steps including transcription, mRNA splicing, hTR and hTERT synthesis and maturation, structural organization of telomerase RNP, nuclear localization of telomerase, post-translational modifications, and recruitment to the telomeres [53]. Epigenetic mechanisms such as chromatin remodeling, DNA methylation and histone modifications for regulation of *hTERT* transcription have also been described [54, 55]. The expression of *hTERT* is also regulated by post-transcriptional mechanisms. The process of gene transcription leads to the generation of transcripts (sequence of pre-mRNA produced by transcription) that are further modified into translational forms by several processes such as mRNA capping (5′-cap), 3′-polyadenylation and alternative splicing. Alternative splicing of *hTERT* mRNA has been shown to be a key post-transcriptional regulatory mechanism [56] but it remains unclear whether telomerase activity is directly associated with *hTERT* splicing.

## Telomerase as a target for anticancer therapeutics

Telomerase has been a prime target for the development of effective therapeutics against cancer as it is expressed in the majority of cancer types as well as in cancer stem or stem-like cells. In addition, normal human cells including stem cells have lower telomerase activity and generally maintain telomeres at longer lengths compared to cancer cells. These features provide an advantage that ensures minimum risk for possible telomere shortening in normal cells. The main objective of anti-telomerase therapeutics is to selectively induce apoptosis and cell death in cancer cells while minimizing the effects on normal cells [57]. Multiple approaches have been adopted to achieve this goal through the development of vaccines, antisense oligonucleotides, and small-molecule inhibitors targeting hTERT or hTR. Although the oligonucleotide imetelstat (GRN163L) appears to be the most promising telomerase inhibitor, Bryan and colleagues [58] have reported a novel telomerase inhibitor, BIBR1532, that binds to the thumb domain of TERT, disrupting TERT–RNA binding (telomerase ribonucleoprotein assembly), leading to the inhibition of enzyme activity. However, this compound has not yet progressed to clinical trials. Additionally, development of G-quadruplex stabilizers, tankyrase (which has an important role in telomere homeostasis, mitotic spindle formation and WNT/β-catenin signaling) inhibitors and HSP90 (involved in signal transduction, intracellular transport and protein degradation) inhibitors targeting telomere and telomerase assembly, and T-oligo (DNA oligonucleotide homologous to the telomere 3′ overhang region, which causes cytotoxic effects by inducing DDR) have also been explored to selectively kill cancer cells [59]. In addition, immunotherapies that use dendritic cells (GRVAC1), hTERT peptide (GV1001) or cryptic peptides (Vx-001) are being tested in clinical trials. Several anti-telomerase agents (imetelstat and vaccines) are currently undergoing different phases of clinical trials but imetelstat is the only anti-telomerase compound that has been extensively evaluated in clinical trials. Recently, the US Food and Drug Administration (FDA) removed a longstanding clinical hold on imetelstat and it is expected to complete planned clinical trials (Table 2).

**Table 2:** Completed and ongoing clinical trials of imetelstat in cancer patients

Identifier code/ phase	Indication	Objective	Start/ completion date	Design	Results	Sponsor
NCT00594126/phase I	Refractory or relapsed multiple myeloma	Safety and MTD determination	November 2007/July 2011	3 + 3 cohort; dose escalation study	DLT: thrombocytopenia, neutropenia, anemia, aPTT prolongation, fatigue, nausea, anorexia and dizziness.	Geron
NCT00732056/phase I	Recurrent or metastatic breast cancer	Safety, MTD, efficacy in combination with paclitaxel and bevacizumab	July 2008/March 2012	3 + 3 cohort;dose escalation study	DLT: thrombocytopenia, neutropenia.	Geron
NCT00310895/phase I	Solid tumor malignancies	Safety and MTD determination	March 2006/March 2013	3 + 3 cohort; dose escalation study	DLT: thrombocytopenia, myelosuppression.	Geron
NCT 00718601phase I	Multiple myeloma	Safety and MTD determination in combination with bortezomib and dexamethasone	July 2008/October 2011	3 + 3 cohort; dose escalation study	Results not available in public domain.	Geron
NCT00124189/phase I	Refractory chronic lymphoproliferative disease	Safety, tolerability, dose-limiting toxicities, and MTD	July 2005/March 2013	Sequential dose cohort, open label, escalation trial evaluating one infusion duration of 2 h; weekly intravenous infusion	Results not available in public domain.	Geron
NCT00510445/phase I	Metastatic non-small-cell lung cancer	Safety, DLT, MTD in combination with a standard paclitaxel/carboplatin regimen	July 2007/April 2011	Dose cohorts with a minimum of 3 patients	Patients on imetelstat with short TL showed a trend towards longer median PFS as well as OS. However, imetelstat treatment in patients with long TL had no improvement in median PFS or OS.ADRs: neutropenia and thrombocytopenia.	Geron
NCT01265927/phase I	HER2^+^ breast cancer	DLT in combination with trastuzumab	January 2011/October 2015	Open label, non-randomized study	Results not available in public domain.	Geron
NCT01242930/phase II	Multiple myeloma	Improved outcome in patients previously treated with imetelstat.	November 2010/December 2015	Imetelstat 2 h intravenous Infusion on day 1 and day 8 of a 28-day cycle	Results not available in public domain.	Geron
NCT02598661/phase III IMerge™	Myelodysplastic syndrome	Safety and efficacy	November 2015/May 2019	Randomized, double blind	Recruiting participants.	Janssen
NCT02426086/phase II IMbark study	Myelofibrosis patients previously treated with JAK inhibitor	Safety and efficacy	June 2015/March 2018	Randomized, single-blind, multicenter	Recruiting participants.	Janssen
NCT01243073/phase II	Essential thrombocythemia	Safety and efficacy	December 2010/April 2015	Open label, single group	Eighteen patients, all with positive hematologic response. Positive molecular response in most patients with *JAK2* V617F mutation. ADRs: neutropenia, anemia.	Geron
NCT01731951/phase II	Myelofibrosis	Efficacy	October 2012/January 2019	Open label, parallel, active, not recruiting	Complete or partial remission in 21 % patients. Bone marrow fibrosis was reversed in a few patients.	Janssen

*ADR* adverse drug reaction, *aPTT* activated partial thromboplastin time, *DLT* dose-limiting toxicity, *MTD* maximum tolerated dose, *OS* overall survival, *PFS* progression-free survival, *TL* telomere length

## Developmental highlights of oligonucleotide inhibitor imetelstat

**Fig. 5:**
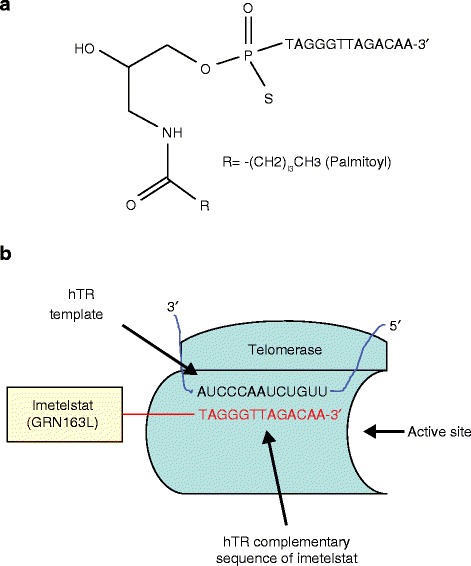
Structure and action of imetelstat (GRN163L). **a** Structure of imetelstat. Imetelstat is a lipid-conjugated 13-mer oligonucleotide sequence with a thio-phosphoramidate backbone. The oligonucleotide sequence is complementary to the hTR component of telomerase and is responsible for the inhibitory activity of imetelstat, whereas the thio-phosphoramidate backbone imparts resistance to the action of plasma and cellular nucleases. **b** Action of imetelstat. Imetelstat binds to the hTR template region at the hTERT active site with high affinity and blocks its recruitment to telomeric DNA. Imetelstat is a competitive telomerase template antagonist (not antisense that targets mRNA). Binding of imetelstat to hTR results in telomerase inhibition leading to progressively shortened telomeres

Imetelstat is a competitive inhibitor of telomerase activity, and was developed for the intravenous treatment of various cancers. It consists of a 13-mer N3′–P5′ thio-phosphoramidate oligonucleotide that is covalently attached to a palmitoyl (C16 lipid) moiety through a 5′-thio-phosphate group (Fig. 5a). The thio-phosphoramidate backbone of imetelstat is responsible for its outstanding features such as high aqueous solubility, acid and metabolic stability, resistance to the action of nucleases, and ability to form RNA duplexes [60]. The lipid moiety of imetelstat provides high lipophilicity that enhances cellular uptake, retention and drug efficacy [61]. Imetelstat does not behave like a typical antisense oligonucleotide as it does not bind to mRNA to inactivate it; rather its sequence (5′-palmitate-TAGGGTTAGACAA-NH_2_-3′) binds to a complementary 13-nucleotide region of hTR that has high affinity and specificity at the active site of the telomerase holoenzyme, thus leading to complete inhibition of enzyme activity (Fig. 5b).

Imetelstat has been extensively evaluated for its activity and efficacy against multiple cancer cell lines and in mouse xenograft models in preclinical studies. Imetelstat demonstrated potent inhibitory action against telomerase, causing shortening of telomeres in a large spectrum of cancer cell lines derived from tumors of the bladder, breast, lung, liver, prostrate and pancreas [62–64]. In vivo preclinical studies in mouse models of human tumor xenografts showed that the compound was well tolerated and highly efficacious in inducing telomerase inhibition, leading to reduced tumor growth, prevention of metastasis, and sensitization of tumors to standard chemotherapy [65]. Imetelstat was also found to efficiently prevent glioblastoma tumor growth in a xenograft model by crossing the blood–brain barrier, probably owing to its highly lipophilic nature [66]. Additionally, simultaneous suppression of homologous recombination and telomerase activity in a mouse model of Barrett’s adenocarcinoma with the combination of nilotinib (tyrosine kinase inhibitor) and imetelstat was reported to be more effective compared to either compound alone [67].

Imetelstat has been undergoing clinical trials for several years, and while some trials have already been completed, some were discontinued (breast and lung cancer, lymphoproliferative disorders and polycythemia vera) because the US FDA put these on hold due to hematological toxicity, but a few are still continuing (Table 2). Recent clinical development of imetelstat includes two studies, one with patients with myelofibrosis, referred to as the Initial MF Study or the IMbark™ study, and one with patients with myelodysplastic syndrome, called the MDS or IMerge™ study (Table 2). Currently, these studies are recruiting targeted patients at various centers in the USA, Europe and Asia.

## Anti-telomerase immunotherapeutics

**Fig. 6:**
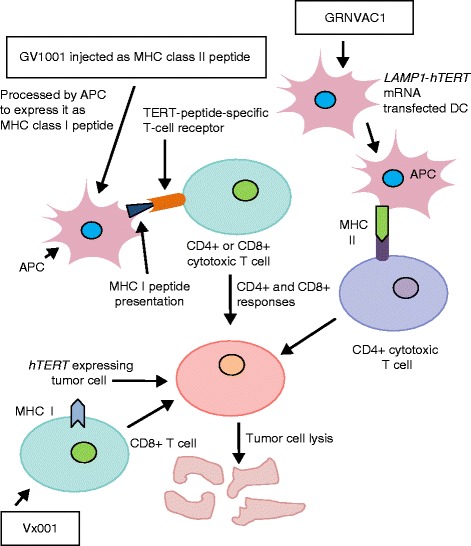
Anti-telomerase immunotherapy. Several telomerase-based vaccines have been developed, which sensitize immune cells to cancer cells expressing hTERT peptides as surface antigens via the human leukocyte antigen (HLA) class I and class II pathways. This results in an expansion of telomerase-specific CD4^+^ and CD8^+^ cytotoxic T lymphocytes (CTLs) in cancer patients leading T cells to target and kill telomerase-positive tumor cells. GV1001 is an MHC class II-restricted hTERT peptide that is further processed by antigen presenting cells (*APCs*) to present as an MHC class I peptide, and it produces both CD4^+^- and CD8^+^-based immune responses. GRNVAC1 stimulates CD4^+^ T cells to target and kill hTERT-expressing tumor cells. Vx001 action is mediated by CD8^+^ T cells

Telomerase is an attractive target for the development of telomerase-based immunotherapy. In cancer cells, the degradation of telomerase by proteasomes results in the formation of protein fragments or peptides of telomerase that are expressed on the tumor cell surface as antigens by the human leukocyte antigen (HLA) class I pathway [68, 69], and these telomerase antigenic epitopes can be targeted by cytotoxic T cells to destroy the tumor cells [70]. Telomerase-specific epitopes can induce CD4^+^ or CD8^+^ cytotoxic T-lymphocyte responses or stimulate antigen-presenting cells capable of attacking tumors [71] (Fig. 6). Therefore, the rationale for anti-telomerase immunotherapy is to sensitize the immune system to tumor cells expressing hTERT peptides to activate and generate hTERT-specific CD8^+^ cells to produce enhanced anti-tumor effects. Two major strategies have been adopted to develop effective telomerase-based immunotherapy in cancer: an hTERT vaccine approach and a dendritic cell approach to prime antigen-presenting cells ex vivo. Three hTERT vaccines, GV1001, Vx001 and GRNVAC1, have been used to elicit anti-telomerase immune responses in cancer patients [72].

GV1001 is a 16-amino-acid, HLA class II-restricted hTERT peptide that contains amino acid sequence 611–626 (EAR-PALLTSRLRFIPK) of the hTERT active site [73]. Granulocyte–monocyte colony-stimulating factor (GM-CSF) or TLR7 is used as adjuvant to carry GV1001. The vaccine is endogenously processed to yield a HLA class I peptide producing both CD4^+^ and CD8^+^ responses, thus evoking strong cytotoxic T-lymphocyte activation [74]. Another vaccine called Vx001 is a cryptic peptide (functional peptides hidden in protein structures)-based vaccine containing hTERT amino acid sequence YLFFYRKSV. The vaccine shows high affinity for HLA class I and has demonstrated a significant immune response rate in cancer patients [75, 76]. A dendritic-cell-based vaccine, GRNVAC1, consists of mature autologous dendritic cells transduced with mRNA encoding hTERT and LAMP1. LAMP1 guides hTERT to lysosomes, where it is degraded into small peptides, leading to a polyclonal immune response specific to all hTERT epitopes expressed by patient tumors [77]. GRNVAC1 was found to be well tolerated with no signs of autoimmunity after three or six weekly injections and elicited robust immune response in patients [78]. Currently, all these vaccines (GV1001, GRNVAC1 and Vx001) are undergoing clinical trials in cancer patients, and the hTERT-specific immune responses elicited by these vaccines were found to be well tolerated in the majority of patients (Table 3).

**Table 3:** Completed and ongoing clinical trials of anti-telomerase vaccines: current status

Identifier code/phase	Indication	Objective	Start/completion date	Results	Sponsor/reference
NCT00510133/GRNVAC1 phase II	Acute myelogenous leukemia	Efficacy	July 2007/August 2014	GRNVAC1 was found to be safe and well tolerated. Positive immune responses in 55 % of patients. Toxicity: thrombocytopenia.	Asterias Biotherapeutics (http://asteriasbiotherapeutics.com/pipeline/ast-vac1/)
NCT01579188/GV1001 phase III	Non-small-cell lung cancer	Efficacy	May 2012/May 2016	Ongoing.	Kael-GemVax
NCT00425360/GV1001 phase III	Metastatic pancreatic cancer	Efficacy in combination with chemotherapy	September 2006/ March 2013	Adding GV1001 vaccination to chemotherapy did not improve overall survival.	[108]
NCT01935154/Vx001 phase II	Non-small-cell lung cancer	Efficacy	August 2012/ December 2016	Active.	Vaxon Biotech (http://www.clinicaltrials.gov)

Clinical trial results have demonstrated that GRNVAC1, Vx001 and GV1001 are promising telomerase-targeting vaccines capable of stimulating CD4^+^ and CD8^+^ responses in telomerase-positive tumors, showing minimal effects on normal cells and no autoimmunity. Large multicenter studies are required to determine long-term toxicities in patients. However, at present, it is not certain if any of these vaccine candidates will progress to registration studies to get approval for clinical application.

## Exploiting telomerase activity to selectively kill cancer cells

A major challenge for anti-telomerase-directed therapy is the long lag period required to observe enough TL attrition to induce cell death. Telomere shortening requires a series of cell division cycles to become apparent, and treatment may have to be given continuously for months to induce therapeutically relevant tumor reduction effects. During this treatment period, most tumor cells will continue to grow, which may require the use of other treatment modalities for successful clinical outcomes. Importantly, with direct telomerase inhibitors, if the patient has hematological or any other toxicities (for example, one concern with imetelstat is the development of hematological toxicities requiring drug holidays), then going off treatment for a few weeks would reverse some of the benefits already obtained—the decision about treatment termination or stopping treatment for a short duration may depend upon the risk–benefit ratio in terms of efficacy and manifested toxicity.

Therefore, novel fast-acting therapeutic agents that can inhibit telomerase activity would be highly desirable. One such strategy is not to target telomerase directly but to introduce a modified nucleoside into cells so that telomerase would preferentially incorporate it into telomeric DNA. An altered nucleotide incorporated into telomeres would not bind to shelterin proteins efficiently and should lead to telomere dysfunction and rapid cell death. Mender and colleagues [79] have recently demonstrated that, in telomerase-positive cells, 6-thio-2′-deoxyguanosine (6-thio-dG), a nucleoside analogue of 6-thioguanine (an approved drug), is recognized by telomerase and incorporated into telomeres. This results in altered telomere organization and activation of telomere-associated DNA damage signals called telomere dysfunction-induced foci, and rapid cell death. The nucleoside analogue 6-thio-dG has been evaluated against cell lines and in vivo. Treatment with 6-thio-dG resulted in rapid cell death, whereas normal telomerase-silent (telomerase-negative) human fibroblasts and normal human colonic epithelial cells were largely unaffected. In in-vivo studies, 6-thio-dG treatment caused significant reduction in tumor growth rates and was superior to 6-thioguanine treatment. Additionally, mice treated with 6-thio-dG at effective doses for a month did not show any hematological, hepatic or renal side effects. Thus, a telomerase-mediated telomere-disrupting approach may provide a safe and efficacious option for the treatment of cancer [80].

## Conclusions and future perspectives

Telomere maintenance has been extensively studied, and our understanding of the role of telomerase and ALT in cancer has improved remarkably in recent years. It is becoming clear how cancer cells regulate different molecular events involved in telomere maintenance to expand their proliferative capacity. Recent insights into the control of telomerase activity at telomeres, through telomerase–shelterin interactions, by regulating telomerase recruitment or productive substrate engagement at the enzyme active site, have highlighted opportunities for the development of novel diagnostic tools and effective anticancer agents. Furthermore, recent knowledge gained about the mechanisms underlying the non-canonical functions of telomerase has significantly improved our understanding of the role of telomerase in cancer progression. However, further research efforts are needed to obtain an in-depth understanding of *hTERT* activation in the initial stages of carcinogenesis, and the various genetic and epigenetic mechanisms involved in its regulation. While the recurrent *hTERT* promoter mutations are highly frequent in many cancers and play a pivotal role in the induction of telomerase reactivation in cancer cells, much remains to be learned about the sufficiency or necessity of *hTERT* promoter mutations in cancer initiation and progression. It is still not established whether telomerase expression has any oncogenic characteristics or is simply required for the maintenance of sustained tumor growth (that is, whether it is permissive). Moreover, there are many other unresolved questions regarding telomeres and telomerase function that deserve further investigation (Box 2). Although target-based compounds have greatly benefited patients who have tumors with specific oncogenic mutations, such as EGFR mutation, HER2 amplification, or mutations resulting in ALK expression or KIT expression, the vast majority of common tumors remain less responsive to these target-based drugs. Therefore, novel targeted interventions are required and telomerase inhibition remains a promising strategy for cancer treatment. Recent advances in telomere biology are beginning to unravel potential new telomerase targets (Box 3) for the design of novel molecules targeting the activity of this key enzyme.

Clinical trials with telomerase inhibitors have established telomerase as a viable target, but the time lag between drug administration and clinical response is long. Continued treatment is required for successful clinical outcome, which may lead to severe toxicity in patients. Therefore, a major challenge is to develop a telomerase inhibitor that rapidly kills telomerase-positive tumor cells while sparing normal telomerase-carrying cells.

## Box 1. Major historical research milestones in telomere and telomerase biology

**Telomeres**: Discovered by American geneticist Hermann J. Muller working on *Drosophila melanogaster* in 1938. He observed that the ends of irradiated chromosomes were resistant to mutagenic X-rays and did not undergo deletions or inversions due to the presence of cap-like structures that he called “telomeres” [81].

**A crucial role for telomeres in chromosomal integrity**: Elucidated by Barbara McClintock in 1941. She described that rupture of the chromosomes resulted in the formation of dicentric chromosomes due to fusion of their ends, and demonstrated that damaged ends of the chromosomes could be restored [82].

**Cellular immortality in culture**: Alex Carrel, recipient of the 1912 Nobel Prize in physiology, working at the Rockefeller Institute demonstrated that chick heart tissue culture cells can be maintained in long-term cultures by replenishing with fresh culture medium. He hypothesized that the lifespan of cultured tissues could be extended indefinitely and that the tissues should intrinsically be able to maintain permanent life in vitro under ideal culture conditions. Later, Carrel's associates showed a continuous culture of chick heart cells from 1912 to 1946, and the idea of cell immortality as an intrinsic property was widely accepted by the scientific community. However, it was then discovered that the use of chick embryo extract to culture these cells was actually re-seeding fetal cells and thus the immortality reported by the Carrel laboratory has been largely discounted.

**The concept of normal cell immortality challenged**: Leonard Hayflick, in 1961 at the Wistar Institute, demonstrated that normal human fetal cells in culture could divide only 40 to 60 times, and after that they underwent aging at the cellular level (then called phase III and now replicative senescence) [83].

**End replication problem**: In 1971, James Watson, the co-discoverer of the DNA double helix, suggested that there was an “end replication problem” due to the mechanism governing semi-conservative DNA replication. Watson predicted, based on the asymmetry of how linear duplex DNA is copied, that each cell division would result in the extreme termini of chromosomes being lost. This would be incompatible with long-term maintenance of the genome owing to progressive chromosome shortening with each replication cycle, eventually reaching a critical point leading to cell senescence or death. In addition, he postulated the existence of a protective mechanism to prevent chromosomal shortening [84].

**Hypothesis about cellular aging**: Also in 1971, Alexsey Olovnikov, a Russian scientist, hypothesized that there could be a problem with the ends of chromosomes. He postulated that progressive shortening of the telomere would eventually run into essential genes, leading to cellular aging and perhaps contributing to human aging [85].

***Tetrahymena thermophila*telomeres tandem repeat sequences**: In 1978, Elizabeth Blackburn and Joseph Gall carried out sequencing experiments for the DNA of the *Tetrahymena thermophila* minichromosome and reported that telomeres contained 20–70 tandem copies of a simple hexanucleotide with the sequence 5′-CCCCAA-3′ on one strand and 5′-TTGGGG-3′ on the complementary strand [86].

**Telomerase**: Blackburn and Carol Greider, at Berkeley in 1985, identified an enzymatic activity capable of extending telomeric sequences. The enzyme was named terminal telomere transferase but is now known as telomerase [87]. Along with Jack W. Szostak they received the 2009 Nobel Prize for their discovery that telomeres are protected from progressive shortening by the enzyme telomerase.

**Human telomerase**: Gregg Morin, in 1989 at Yale University, was the first to report telomerase activity in crude HeLa cell extracts. He also demonstrated that human telomeres consisted of the repeated sequence TTAGGG [88]. In 1994, Jerry Shay and colleagues showed telomerase activity in ~90 % of human cancers and cell lines [89], and in 1998 the same team demonstrated that introduction of *hTERT* (the catalytic protein reverse transcriptase component of telomerase) into normal human cells was sufficient to immortalize cells [90].

## Box 2. Key outstanding research issues in cancer telomere and telomerase biology

**Determining mechanism(s) of escape from crisis**: Cells in crisis undergo tremendous genomic instability due to bridge–fusion–breakage cycles. However, many molecular details remain unclear. What are the molecular features of cells that escape crisis? Do they have *hTERT* promoter mutations? Why do incipient cancer cells during crisis acquire stem cell-like properties?

***hTERT*promoter mutations**: Some believe that *hTERT* promoter mutations drive carcinogenesis, while others believe that promoter mutations are only permissive for tumor growth maintenance. How these widespread *hTERT* promoter mutations regulate *hTERT* expression during cellular transformation is not fully understood.

**Telomerase holoenzyme assembly**: Although there has recently been progress on determining the yeast and ciliate telomerase structure, the processes of assembly and function of telomerase in human cancer cells remain poorly understood.

**Recruitment of human telomerase to telomeres in cancer cells**: The recruitment of telomerase to telomeres is highly regulated and occurs only after the replication fork remodels protected DNA 3′ ends during the S phase of the cell cycle. It involves protein–protein interactions between the shelterin complex components TPP1 and POT1 and the DAT domain of hTERT. TPP1 contains an N-terminal OB-fold domain containing a patch of amino acids termed the Tel patch that directly interacts with the telomerase DAT domain [40]. However, the signaling pathways that regulate telomerase recruitment in human cancer cells are not clearly understood. The telomerase recruitment process is likely regulated by as yet unknown signaling pathways.

**Shelterin protein complex**: Emerging evidence suggests a crucial role for shelterin components in cancer progression, but how these components are regulated during different stages of cancer development is not well understood.

**Alternative lengthening of telomeres**: Recently, knowledge about ALT has increased significantly. The chromatin remodeling factor ATRX acts as a suppressor of ALT in normal cells and mutations in ATRX and DAXX contribute to activation of ALT [28, 91]. However, knockdown of ATRX is not sufficient to trigger the ALT pathway in telomerase-positive cell lines or to directly activate ALT in normal somatic cells, implying the existence of other necessary contributing factors involved in activation of ALT in cancer cells [92, 93]. Thus, many key questions remain unanswered, such as why ALT is more frequent in certain cancer subtypes? How does ATRX/DAXX repress ALT and what is the molecular basis of its activation in cancer cells with wild-type ATRX/DAXX? What is the function of variant DNA repeats in ALT? How does RAD51 interact with the 5′ overhang of ALT telomeric DNA to facilitate its invasion into homologous DNA, and how are shelterin proteins organized in ALT telomeres? Answers to these questions may facilitate development of mechanism-based inhibitors for ALT-positive cancers.

## Box 3. Telomere biology-based potential novel targets for the development of anticancer agents

**Inhibition of the Tel patch to block telomerase recruitment to telomeres**: The “Tel patch”, a specific amino acid sequence in the OB-fold domain of shelterin complex protein TPP1, is involved in telomerase binding, recruitment, enzyme processivity and telomere elongation. Thus, inhibition of telomerase recruitment may result in cell death [94].

**Inhibition of telomerase non-canonical function mediators**: In addition to telomere maintenance, telomerase may also be involved in other important activities such as regulating gene expression, mitochondrial activity, cell proliferation, apoptosis, epithelial–mesenchymal transitions and DNA damage repair. These non-canonical putative telomerase functions may be mediated through a network of “feed forward signaling loops” [95]. Interventions targeting the molecules involved in non-telomeric functions of telomerase may be a rational approach for cancer treatment.

**Inhibition of TRF1 shelterin protein**: TRF1 is overexpressed in many cancer types and plays a central role in controlling replication of telomeric DNA. The genetic abrogation of TRF1 leads to a marked reduction in lung carcinoma tumor growth in the *K-Ras*^*G12V*^ lung cancer mouse model due to acute telomere uncapping independent of telomere length [96]. However, it is not clear what effects targeting shelterin proteins would have on normal cells.

**Inhibition of ATM kinase**: The ATM kinase plays a crucial role in the cellular response to telomere dysfunction-mediated DNA damage and subsequent repair pathways. ATM has recently been shown to be required for the addition of telomeric DNA repeats to telomeres and telomere elongation by telomerase in human cells. Blocking ATM inhibits telomere elongation and inhibition of PARP1, which activates ATM and increases telomere elongation [97]. ATM may regulate telomerase access to telomeres through interaction with TRF1 [98].

**Inhibition of alternative splicing of mRNA of*hTERT***: The human *TERT* gene produces numerous alternatively spliced variants with a few isoforms capable of producing full-length catalytically active telomerase. A fuller understanding of the process of alternative splicing may lead to the development of molecules to inhibit the generation of full-length telomerase and be a new approach to telomerase therapy in cancer [56].

**Inhibition of TERT RNA-binding domain (tTRBD)**: TERT protein binds to the template boundary element of TR (TERC), crucial for the recognition of the precise telomere sequence to be reverse transcribed by *TERT* [99]. This is a potential intervention target, but this discovery needs to be established in human cells.

## Abbreviations

ALT, alternative lengthening of telomeres; bp, base pairs; DAT, dissociates the activities of telomerase; DDR, DNA damage response; FDA, Food and Drug Administration; GM-CSF, granulocyte–monocyte colony-stimulating factor; hESC, human embryonic stem cell; kb, kilobases; OB, oligonucleotide/oligosaccharide-binding; TERRA, telomeric repeat-containing RNA; T-loop, telomeric loop; TL, telomere length; TPE, telomere position effect; TSS, transcription start site
